# Should oral foci of infection be removed before the onset of radiotherapy or chemotherapy?

**DOI:** 10.1111/odi.13329

**Published:** 2020-06-01

**Authors:** Fred K. L. Spijkervet, Jennifer M. Schuurhuis, Monique A. Stokman, Max J. H. Witjes, Arjan Vissink

**Affiliations:** ^1^ Department of Oral and Maxillofacial Surgery University Medical Center Groningen University of Groningen Groningen The Netherlands; ^2^ Department of Radiotherapy University Medical Center Groningen University of Groningen Groningen The Netherlands

**Keywords:** chemotherapy, head and neck oncology, hematology, oral foci of infection, radiotherapy, teeth

## Abstract

Pretreatment dental screening aims to locate and eliminate oral foci of infection in order to eliminate local, loco‐regional, or systemic complications during and after oncologic treatment. An oral focus of infection is a pathologic process in the oral cavity that does not cause major infectious problems in healthy individuals, but may lead to severe local or systemic inflammation in patients subjected to oncologic treatment. As head and neck radiotherapy patients bear a lifelong risk on oral sequelae resulting from this therapy, the effects of chemotherapy on healthy oral tissues are essentially temporary and reversible. This has a large impact on what to consider as an oral focus of infection when patients are subjected to, for example, head and neck radiotherapy for cancer or intensive chemotherapy for hematological disorders. While in patients subjected to head and neck radiotherapy oral foci of infection have to be removed before therapy that may cause problems ultimately, in patients that will receive chemotherapy such, so‐called chronic, foci of infection are not in need of removal of teeth but can be treated during a remission phase. Acute foci of infection always have to be removed before or early after the onset of any oncologic treatment.

## 1 INTRODUCTION

Pretreatment dental screening aims to locate and eliminate oral foci of infection in order to prevent local, loco‐regional, or systemic complications during and after oncologic or other medical treatments (Beech, Robinson, Porceddu, & Batstone, 2014; Eliyas, Al‐Khayatt, Porter, & Briggs, 2013; Hong et al., 2010; Schuurhuis et al., 2015). An oral focus of infection is defined as a pathologic process in the oral cavity that does not cause major infectious problems in healthy individuals, but may lead to severe local or systemic inflammation under certain circumstances (Nabil & Samman, 2011; Sennhenn‐Kirchner et al., 2009).

Frequently observed potential oral foci of infection include caries profunda, periodontal disease, periapical problems, (partially) impacted or partially erupted teeth not fully covered by bone or showing radiolucency, cysts, non‐vital pulps, and ulcerations (Ben‐David et al., 2007; Jansma et al., 1992; Schuurhuis et al., 2015; Stokman, Vissink, & Spijkervet, 2008). However, which pathologic oral process should be considered as an oral focus of infection is dependent on the underlying medical problem. For example, patients with an oral squamous cell carcinoma treated with curative ionizing radiation therapy to the head and neck region possess a lifelong risk to develop treatment‐related sequelae, such as osteoradionecrosis (ORN) of the jaws (Chrcanovic, Reher, Sousa, & Harris, 2010; Nabil & Samman, 2012; Spijkervet, Brennan, Peterson, Witjes, & Vissink, 2019). Therefore, it is commonly accepted, although not always evidence‐driven, that such patients have to be free of oral foci of infection 10–14 days before the onset of radiotherapy to allow possible tooth extraction wounds to heal (Beech et al., 2014; Jansma et al., 1992). On the contrary, the effects of chemotherapy on healthy oral tissues are essentially temporary and reversible. Thus, the risk of developing complications related to chronic oral foci of infection is probably not higher than in healthy subjects once patients have recovered from chemotherapy and their blood levels have normalized.

This review will (a) address what is considered an oral focus of infection and (b) propose guidelines on managing these foci of infection prior to cancer treatment of head and neck cancer or hematologic diseases.

## 2 HEAD AND NECK CANCER

Radiotherapy to the head and neck region involving the oral cavity and salivary glands results in multiple acute and late side effects such as oral mucositis, a reduced salivary flow (hyposalivation), a sensation of oral dryness (xerostomia), loss of taste, trismus, skin fibrosis, and an increased risk of developing dental caries and fungal and bacterial infections (Jensen, Vissink, Limesand, & Reyland, 2019; Nabil & Samman, 2012; Vissink, Jansma, Spijkervet, Burlage, & Coppes, 2003). The main reason for dental screening on oral foci of infection in head and neck cancer (HNC) patients is to prevent acute and long‐term oral sequelae, especially ORN. Comparison of the data on ORN reported in the literature is hard as no unambiguous definition of ORN is applied which may result in under‐ or overreporting of ORN. For example, many patients may have low‐grade jaw complications, such as exposed bone, which is not reported as ORN (Beadle et al., 2013).

In the last decades, radiation treatment of HNC has changed substantially, among others due to the introduction of intensity‐modulated radiation therapy (IMRT) and concomitant chemoradiation (CHIMRT) (Bortfeld, 2006; Grégoire, Langendijk, & Nuyts, 2015; Schwartz & Hayes, 2020). The exact effects of IMRT on the oral microflora, oral tissues, and jaw bone are not yet clear. Neither which oral foci of infection have to be considered an oral focus of infection needing treatment before the onset of IMRT. For example, it has been shown that IMRT results in less xerostomia due to sparing of the parotid and/or submandibular glands (Jellema, Slotman, Doornaert, Leemans, & Langendijk, 2007; Jensen et al., 2019; Vissink, van Luijk, Langendijk, & Coppes, 2015; Vissink et al., 2010). But at the same time, sparing of, for example, salivary glands may result in higher doses to the other tissues in the radiation field, such as the jaw bone (Hansen et al., 2012). Higher doses to jaw bone bear a higher risk of developing ORN. Also, the role of the oral microbiome in the development, treatment response, and treatment toxicity in patients with HNC needs to be further explored (Orlandi et al., 2019).

### 2.1 Osteoradionecrosis

The effects of radiation on salivary glands (hyposalivation) and bone (ORN) are the side effects of radiotherapy with the greatest impact on the patients’ quality of life. While hyposalivation and its related effects are difficult to prevent and are usually at best reduced to a minimum, ORN is probably easier to prevent or at least the risk of developing ORN can be minimized (Spijkervet et al., 2019). Schuurhuis et al. (2011) showed in their retrospective study that patients presenting with severe periodontal disease at dental screening are prone to develop ORN, particularly when periodontally affected teeth are not (aggressively) treated. Next, in a 2‐year prospective study Schuurhuis et al. (2018) indeed showed that patients with periodontal disease before IMRT/CHIMRT were prone to develop bone healing problems after IMRT/CHIMRT. Due to the less reduced salivary flow rate seen after IMRT compared to conventional radiotherapy (Jensen et al., 2019; Vissink et al., 2015), the risk of developing rapidly progressing dental caries may be reduced. As a result, teeth will be longer preserved in IMRT patients, increasing the hazard of developing periodontitis and thus the hazard of developing ORN during long‐term follow‐up.

As mentioned, the oral microbiome might play a role in the development, treatment response, and treatment toxicity in patients with HNC (Gaetti‐Jardim et al., 2018; Orlandi et al., 2019). In this respect, it is important to note that Schuurhuis, Stokman, et al. (2016) found an almost immediate effect on the composition of the oral flora after the elimination of oral foci of infection, with a decrease of periodontal pathogens. However, rather high percentages of periodontal pathogens were present after 1 year of follow‐up in patients with a history of periodontal disease, which may underly the progression of pocket depth observed.

### 2.2 What to consider as an oral focus of infection in HNC patients?

The evidence of the efficacy of elimination of oral foci of infection to prevent postradiotherapy oral sequelae is growing (Beech et al., 2014; Eliyas et al., 2013; Jansma et al., 1992; Muraki et al., 2019; Schuurhuis et al., 2011, 2018; Sennhenn‐Kirchner et al., 2009), and in particular what to consider as an oral focus of infection in specific patient groups. We suggest, based on the literature, that the following should be considered as an oral focus of infection in HNC patients:

- deep caries in which excavation may lead to pulpal exposure;
- active periodontal disease with pockets ≥ 6 mm, furcation > grade 1, mobility > grade 1, gingival recession ≥ 6 mm, and especially a combination of these periodontal problems;
- non‐restorable teeth with large restorations, especially those extending the gum line or with root caries;
- periapical granuloma and avital teeth;
- (partially) impacted or partially erupted teeth not fully covered by bone or showing radiolucency;
- cysts and other radiographic abnormalities.

While in hematology patients the effects of chemotherapy are mainly reversible, the effects of radiotherapy are mainly irreversible and the risk to develop complications remains a lifelong.

### 2.3 Preradiotherapy dental screening guidelines in HNC patients

Although no strict guidelines for preradiotherapy dental screening and elimination of oral foci exist, recent studies in HNC patients have shown that a strict execution of a dental screening protocol is mandatory (Bichsel, Lanfranchi, Attin, Grätz, & Stadlinger, 2016; Muraki et al., 2019; Schuurhuis et al., 2018). Not aggressively treating periodontally affected teeth preradiotherapy results in an increased risk for ORN, and patients with periodontal disease before IMRT are prone to develop bone healing problems after IMRT. Furthermore, progression of periodontal pocket depth has been reported in HNC patients treated with IMRT/CHIMRT (Schuurhuis et al., 2011,2016,2018).

The patients’ periodontal status at dental screening and the probability of progression of periodontal disease after IMRT/CHIMRT should be considered carefully in dental treatment planning before radiotherapy as periodontal disease is linked to a higher risk of developing ORN after radiotherapy. A strict execution of a well‐defined dental screening protocol is likely to result in fewer postradiotherapy extractions and, therefore, less ORN since postradiotherapy extractions are a well‐known risk factor (Nabil & Samman, 2011, 2012). Although there is no literature available evaluating the economic impact of ORN (Peterson et al., 2010), high costs are inevitable when, for example, surgical intervention and hyperbaric oxygen therapy are mandatory (Spijkervet et al., 2019). Reducing the incidence of ORN is likely to reduce healthcare costs and, more importantly, may prevent unneeded suffering from the patients and thus improve quality of life.

It is recommended to perform a preradiotherapy dental screening for HNC patients subjected to radiotherapy according to an (as far as available) evidence‐based protocol, for example, the protocol (Table 1) used by Jansma et al. (1992) and Schuurhuis et al. (2018). This screening should preferably be done at least 10–14 days before the start of radiotherapy to allow for healing of, for example, extraction sites, before radiotherapy is started. The dental screening should take into account the expected radiation dose to the jaws. What the lowest tolerable dose exactly is, has never been proven. The risk of developing ORN starts approximately at 40 Gy and increases with increasing dose, with cumulative radiation doses >60 Gy creating a high risk when foci have not been eliminated (Spijkervet et al., 2019).

**Table 1:** Assessment and treatment of oral foci of infection within or outside the radiation field (Schuurhuis et al., 2018)

Assessed tooth problems	Treatment if cumulative dose >40 Gy^a^	Treatment if cumulative dose <40 Gy or outside the radiation portal >40 Gy^a^
Caries profunda	Tooth extraction	Restoration, if necessary combined with endodontic treatment, or tooth extraction
Periapical pathosis (on radiographs) without symptoms and/or additional problems	*In teeth without root canal filling*: Endodontic treatment and/or apexification *In teeth with root canal filling*: Endodontic re‐treatment, apexification, or tooth extraction (needed in case of preradiotherapy time limitations)	*In teeth without root canal filling* : Endodontic treatment *In teeth with root canal filling*: Endodontic re‐treatment, apexification, or tooth extraction Treatment can be postponed until after radiotherapy
Extensive periapical pathosis (on radiographs) combined with periodontal disease, in afunctional teeth or *with symptoms*	Tooth extraction	*In teeth without root canal filling*: Endodontic treatment combined with initial periodontal treatment *In teeth with root canal filling*: Endodontic re‐treatment, apexification, or tooth extraction depending on the prognosis
Avital pulp *with symptoms* and without periapical radiolucency on radiographs	Endodontic treatment or tooth extraction (which might be necessary in case of preradiotherapy time limitations)	Endodontic treatment or tooth extraction depending on the prognosis
Avital pulp *without symptoms* and without periapical radiolucency on radiographs	Endodontic treatment or tooth extraction (needed in case of preradiotherapy time limitations)	Endodontic treatment (which can be postponed until after radiotherapy)
*Periodontal disease with*: Pockets 4−5 mm Pockets ≥6 mm Gingival recessions ≥6 mm	Initial periodontal therapy Tooth extraction Tooth extraction	Initial periodontal therapy Initial periodontal therapy Only recession requires no treatment
Impacted teeth or roots fully covered by bone without radiographic abnormalities	No treatment If problems are expected in the future: tooth extraction	No treatment
Impacted teeth or roots *not fully covered* by bone or with radiographic abnormalities (e.g., cysts, apical radiolucency)	Tooth extraction	No treatment or, in case of symptoms, surgical removal Roots with periapical radiolucency might be worth preserving by endodontic treatment and restoration (which can be postponed until after radiotherapy)
Cysts	Surgical removal	Surgical removal
Internal or external root resorption	Tooth extraction	Endodontic treatment or tooth extraction depending on the prognosis

^a^ If an irradiated patient needed treatment, radiation fields were always verified with the Department of Radiation Oncology, and depending on the dose in the specific region where treatment was needed, antibiotic prophylaxis was given to the patient.

Additionally, oral hygiene instructions should be given. Especially, patients with periodontal disease have to be evaluated carefully as periodontal disease is probably a condition making subjects prone to develop ORN. Tooth loss and greater periodontal attachment loss occur in teeth that are included in high‐dose radiation portals (Epstein, Lunn, Le, & Stevenson‐Moore, 1998). Thus, the starting point of a preradiotherapy dental screening should be that, considering the patients’ oral hygiene, motivation and physical abilities, and the risk of developing ORN, it is achievable to maintain teeth. A careful, standardized oral follow‐up with repeated oral hygiene instructions is needed after radiotherapy as a neglect of the required minimum level of oral hygiene poses the patient at a high risk of developing dental caries and oral infections which may need removal of teeth and thus increase the risk for ORN.

## 3 HEMATOLOGIC PATIENTS

Patients undergoing intensive chemotherapy are prone to develop, often reversible, oral side effects, such as oral mucositis, xerostomia, taste changes, and local and systemic infections (Brennan, Elting, & Spijkervet, 2010). Intensive or high‐dose chemotherapy given to hematologic patients causes severe neutropenia (absolute neutrophil count <500/µl), which puts patients at high risk of infections, sepsis, and septic shock (Walsh, 2010). However, once chemotherapy has ended, neutrophil counts return to normal levels thereby reducing the risk of developing oral complications related to oral foci of infection to that of healthy subjects. In this respect, it is important to mention that Kishimoto et al. (2017) recently showed that intensity and duration of neutropenia is linked to oral mucositis and not to odontogenic infection during high‐dose chemotherapy for hematological malignancies. In line with this observation, Sultan et al. (2017) showed that oral health status was not associated with risk of bacteremia. Finally, it has to be noted that in hematologic patients undergoing high‐dose chemotherapy and allogenic stem cell transplant, oral complications may last longer and be of a different kind due to graft‐versus‐host disease (Haverman et al., 2014). The latter oral complications are, however, mainly mucosa‐driven with lichen planus‐like lesions commonly seen in patients with chronic graft‐versus‐host disease (Hull et al., 2015; Imanguli, Pavletic, Guadagnini, Brahim, & Atkinson, 2006). Dental problems that occur in these patients can be treated without an increased risk of developing hard‐to‐treat complications such as ORN in HNC patients.

Thus, the efficacy of dental screening for oral foci of infection in intensively treated chemotherapy patients is questionable: Do acute and chronic oral foci of infection indeed have to be removed before the onset of therapy or can the treatment of certain chronic oral foci of infection be postponed until after treatment?

### 3.1 What to consider as an oral focus in intensively treated hematologic patients

In hematologic patients, a distinction should be made between acute and chronic oral foci of infection. An oral focus of infection is considered as acute if oral pathology and/or teeth cause pain or other symptoms, and chronic if that focus is not exacerbated and is asymptomatic during the previous 3 months before the onset of cancer therapy. Acute oral foci of infection should be eliminated, preferable before the onset of chemotherapy. Schuurhuis, Span, et al. (2016) showed that chronic oral foci of infection can be left untreated in hematology patients subjected to intensive chemotherapy, as this does not increase infectious complications during intensive chemotherapy. Schuurhuis, Span, et al. (2016) also showed that what to consider as an oral focus of infection is dissimilar in chemotherapy patients than in patients submitted to radiotherapy. The reasons underlying this dissimilarity were discussed before.

Unlike previous studies that focused on acute conversions of previously diagnosed chronic dental disease (Toljanic, Bedard, Larson, & Fox, 1999), the study of Schuurhuis, Span, et al. (2016) focused on systemic complications of chronic oral foci of infection. Based on the outcomes of the prospective study of Schuurhuis, Span, et al. (2016), a prechemotherapy dental screening and treatment of oral foci of infection in intensively treated leukemic patients and patients subjected to high‐dose chemotherapy and/or autologous stem cell therapy is recommended.

### 3.2 Guidelines of dental screening in hematologic patients receiving intensive chemotherapy

Although no strict guidelines for prechemotherapy dental screening and elimination of oral foci exist, the study of Schuurhuis, Span, et al. (2016) showed that a less aggressive approach can be executed in leukemic patients subjected to intensive chemotherapy and in multiple myeloma patients, non‐Hodgkin lymphoma patients, and Hodgkin lymphoma patients subjected to high‐dose chemotherapy and autologous stem cell therapy. From their studies, the guideline is as follows: Ask and examine patients oral structures if they had any symptoms like pain, percussion or palpation tenderness, fever, swelling, and/or tooth‐related purulent drainage that might be caused by a pathology related to the oral mucosal and/or dental hard tissues during the past 3 months. When such problems are present, these acute oral problems and pathologies should be eliminated before the onset of chemotherapy. Such an approach is likely to be beneficial for hematologic patients, as removal of teeth may compromise nutrition, and malnutrition is associated with a lower quality of life (Jager‐Wittenaar et al., 2011). Tooth extraction directly before the start of intensive chemotherapy also leads to a risk for infection, bleeding, or delayed wound healing, which may require postponing oncologic treatment (Yamagata et al., 2006), or otherwise increase bacteremia with a higher chance of septic complications. For survivors, treatment of diseased teeth can be postponed until oncologic treatment is completed and neutrophil levels have normalized. Moreover, prechemotherapy dental work‐up will be less time‐consuming and therefore less expensive, when only acute oral foci of infection, seen in <10% of patients who need intensive chemotherapy treatment, have to be treated instead of all the chronic oral foci seen in over 70% of our patients scheduled for intensive chemotherapy (Schuurhuis, Span, et al., 2016).

It is recommended to perform pretreatment dental screening in leukemic patients subjected to intensive chemotherapy and in multiple myeloma, non‐Hodgkin, and Hodgkin patients subjected to high‐dose chemotherapy and autologous stem cell therapy and to carefully consider the need for dental treatment (Table 2). Chronic oral foci of infection can be left untreated until the hematologic treatment has been completed, while acute oral foci of infection should be eliminated, unless there is insufficient time to heal before the start of chemotherapy, knowing that due to the cancer itself, wound healing may already be impaired. When an acute focus of infection cannot be removed before the onset of chemotherapy due to a lack of time, these acute foci of infection have to be removed during the next remission phase. Pretreatment oral hygiene instructions should be given since maintaining a good oral hygiene during chemotherapy is extremely important.

**Table 2:** Assessment and treatment of oral foci of infection in hematologic patients subjected to high‐dose chemotherapy^a^

Oral foci of infection with acute signs or symptoms have to be eliminated before the start of chemotherapy or in an early remission phase
Oral foci of infection (for a listing, see Table 1: assessed tooth problems) without acute signs or symptoms (no exacerbation during the previous 3 months) can be left untreated and can be treated after completion of oncologic treatment

^a^ For a listing of oral foci of infection, see Table 1: assessed tooth problems.

## 4 EPILOGUE

While in patients subjected to head and neck radiotherapy oral foci of infection that may cause problems on the long run have to be removed before therapy, in patients that will receive chemotherapy such, so‐called chronic, foci of infection are not in need of removal of teeth but can be treated during a remission phase. Acute foci of infection always have to be removed before or early after the onset of any oncologic treatment. These recommendations can be deducted from the studies reported in the literature thus far. Many studies yet published in the literature and used to write this review, however, were of a retrospective origin. Moreover, it was often not well‐defined in these studies what to consider oral foci of infection, how dental screening was done, what the tumor characteristics were, as well as the dental history and dental status at the time of dental screening. Thus, the evidence for the recommendations with regard to the removal of oral foci of infection is unfortunately yet not very strong and is in need of well‐defined prospective studies, both in head and neck radiotherapy and hematologic patients.

## CONFLICT OF INTEREST

F.K.L. Spijkervet declares, on behalf of all authors, that we have no conflict of interest.

## AUTHOR CONTRIBUTIONS

F. K. L. Spijkervet design of the review, critical review of the literature, draft writing, approval of the final manuscript. J. M. Schuurhuis literature search, critical review of the literature, critical review of the draft, approval of the final manuscript. M. A. Stokman literature search, critical review of the literature, critical review of the draft, approval of the final manuscript. M. J. H. Witjes critical review of the draft, approval of the final manuscript. A. Vissink design of the review, critical review of the literature, draft writing, approval of the final manuscript.
